# Hyperbaric Oxygen Therapy Alleviates Social Behavior Dysfunction and Neuroinflammation in a Mouse Model for Autism Spectrum Disorders

**DOI:** 10.3390/ijms231911077

**Published:** 2022-09-21

**Authors:** Inbar Fischer, Sophie Shohat, Gilad Levy, Ela Bar, Sari Schokoroy Trangle, Shai Efrati, Boaz Barak

**Affiliations:** 1 The Sagol School of Neuroscience, Tel Aviv University, Tel Aviv 69978, Israel; 2 Department of Biomedical Engineering, Tel Aviv University, Tel Aviv 69978, Israel; 3 The School of Psychological Sciences, Tel Aviv University, Tel Aviv 69978, Israel; 4 The School of Neurobiology, Biochemistry & Biophysics, Faculty of Life Sciences, Tel Aviv University, Tel Aviv 69978, Israel; 5 The Sagol Center for Hyperbaric Medicine and Research, Shamir Medical Center (Assaf Harofeh), Beer Yaakov 70330, Israel

**Keywords:** autism spectrum disorder, Phelan McDermid syndrome, hyperbaric oxygen therapy, neuroinflammation, social behavior, hypoperfusion, Shank3, neurodevelopmental disorders, microglia, Igf-1, Hif1a

## Abstract

Autism spectrum disorder (ASD) is a multifactorial neurodevelopmental disorder (NDD) characterized by impaired social communication and repetitive behavior, among other symptoms. ASD is highly heritable, with *SHANK3* being one of the high-risk genes for ASD. In recent years, knowledge has been growing regarding the neuroplasticity effect induced by hyperbaric oxygen therapy (HBOT) and its potential use for ASD. Here, we characterized the effect of HBOT on a mouse model for ASD with the human genetic condition of InsG3680 mutation in the *Shank3* gene. As compared to placebo, HBOT improved social behavior and reduced neuroinflammation in the cortex of the InsG3680^(+/+)^ mice. Specifically, HBOT induced upregulation of Insulin-like growth factor 1 (*Igf1*) expression levels and reduced the number of Iba1-positive cells in the mouse model for ASD compared to placebo control. Together, our research suggests that HBOT has the potential to improve the clinical outcome of ASD by ameliorating some of the core pathophysiological processes responsible for the development of the disorder.

## 1. Introduction

Autism spectrum disorders (ASDs) are neurodevelopmental disorders (NDDs) characterized by impaired social behavior and communication, restricted interests, and repetitive patterns of behavior, among other symptoms [1]. According to the Centers for Disease Control and Prevention, 1 out of 44 children is diagnosed with ASD in the United States of America, making it one of the most common NDDs [2]. Regarding genes known today, up to 25% of ASD cases have a genetic cause [3]. The focus of this study is on the *SHANK3* gene, which is one of the high-risk genes for monogenic ASD [1,4]. Micro-deletion and variants of *SHANK3* are known to cause Phelan McDermid syndrome (PMS), characterized by autistic-like phenotype, developmental delays, hypotonia, and other symptoms [5].

SHANK3 is a protein that takes part in the postsynaptic density (PSD) of glutamatergic synapses [4]. It is a scaffold protein whose main role is to bind glutamate receptors to the cytoskeleton and therefore is essential for synaptic transmission and integrity [1,4,6]. One of the human mutations found in *SHANK3* is an insertion of guanine in position 3680 of the *SHANK3* gene [6], which causes a frameshift and the appearance of an early stop codon, resulting in a dramatic downregulation of the SHANK3 expression level compared to controls [6,7]. Based on this human genetic condition, the InsG3680 mouse model for ASD was previously characterized [8,9,10], demonstrating major deficits in cortico-striatal synaptic transmission, together with autistic-like behaviors [7].

Although the mutation’s effect on synaptic dysfunction is well-established in the InsG3680 mouse model [7], its effect on glial cells is not well understood. In previous studies, it was shown that synaptic dysfunction could lead to microglia activation, which in turn may alter neuronal communication [11,12,13]. Hyperactivation of microglia could result in chronic neuroinflammation that may affect other properties, such as angiogenesis [14]. In addition, indications of neuroinflammation and impaired angiogenesis were found in various ASD cases in previous studies [15,16]. Therefore, those pathological impairments could play an essential role in the pathophysiology of ASD [17].

Since there is a wide clinical spectrum of symptoms and pathophysiological heterogeneity among patients with ASD [18,19,20], no effective biological intervention was found to target the core pathology, and most treatments used today rely on behavioral intervention and training. Current pharmacological drugs given to patients with ASD include selective serotonin reuptake inhibitors, opioid antagonists, and psychostimulants [21]. Although some treatments are effective, they treat only certain aspects and symptoms of autism but do not address the pathology cure.

One of the well-defined deficits in brain physiology related to ASD is hypoperfusion [15,22] and the consequence of cerebral hypoxia. Based on this physiological deficit, hyperbaric oxygen therapy (HBOT) has the potential to be a beneficial and appropriate treatment to alleviate hypoperfusion [23] and thus improve brain functionality and behavioral outcomes. Moreover, evidence has been accumulated about the neuroplasticity effects of HBOT [24,25,26,27]. HBOT involves the application of hyperbaric pressure in conjunction with increased oxygen content enabling better oxygen diffusion to mal-perfused tissue. It is now understood that the combined action of both hyperoxia and hyperbaric pressure, with oxygen fluctuations generated by the newly used protocol, may target both oxygen and pressure-sensitive genes by the so-called “Hyperoxic-hypoxic paradox” [28]. The fluctuation in oxygen delivery can improve mitochondrial metabolism and induce genes related to stem cell proliferation and angiogenesis [28,29].

The beneficial effects of HBOT were studied mostly in regenerating previously healthy brain tissue exposed to acute insult, while the data on the beneficial effects in brain function abnormalities related to specific born-related DNA mutations are still lacking. Regarding the use of HBOT for ASD, the data are still conflicting, using different protocols and variable cohorts that are not focused on a specific pathophysiological-related subtype of ASD [15,30,31,32,33,34,35,36,37]. One of the patients treated at the Sagol center for hyperbaric medicine, Shamir medical center in Israel, who had ASD, presented a significant clinical improvement in ASD-related behavioral symptoms. This female patient was found to have the InsG3680 mutation in the *SHANK3* gene (unpublished data). Single photon emission computed tomography (SPECT) results before HBOT showed impaired brain perfusion in the orbital-frontal region, dorsa-lateral cortex, medial area of temporal lobes, and in the cerebellum. SPECT analysis after HBOT showed improved brain perfusion and function in the right orbital-frontal cortex, right motor cortex, left frontal temporal lobe, Broca’s area, left dorsal-lateral and dorsal-medial frontal cortex, left occipital lobe, right thalamus nucleus, and the cerebellum compared to the pre-HBOT state. Additionally, the patient’s parents self-reported an improvement in the patient’s social behavior and cognitive performance (these aspects were not measured quantitatively and are not presented).

Motivated by the beneficial outcomes HBOT had on ASD pathology, this study aimed to evaluate the molecular and behavioral effects of HBOT on the InsG3680 mouse model, to better understand the mode of operation HBOT has on the autistic brain.

## 2. Results

### 2.1. HBOT Improves Social Novelty Preference but Not Anxiety-like Behavior and Motor Coordination in InsG3680 Mouse Model for ASD

The physiological and behavioral improvements demonstrated in the human HBOT study prompted us to study the neurobiological properties of HBOT on a mouse model for ASD that harbors the same insertion mutation in *Shank3* as in the human patient. Our motivation was to test whether the improved behavior in the autistic human patient could also be found when proper statistical power is tested, and to study neurobiological properties that can explain the improved behavior reported.

**Figure 1:**
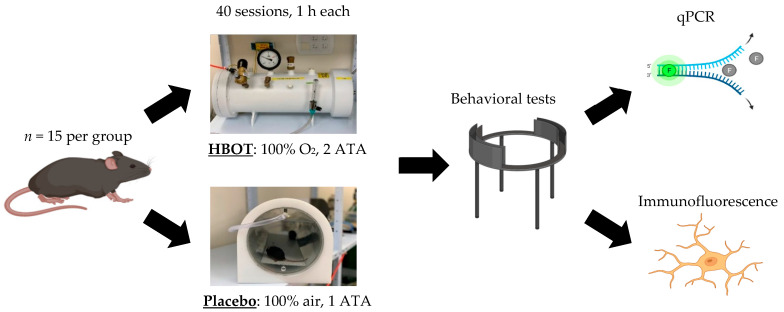
Graphical description of experimental procedures. InsG3680^(+/+)^ and littermates control mice were divided into two groups, HBOT and placebo. After two months of treatment, mice were tested in various behavioral tests, and afterward, InsG3680^(+/+)^ mice were used for qPCR and immunofluorescence analysis.

To achieve this, we studied the effects of HBOT (100% oxygen gas, 2 atmospheres absolute [ATA] pressure levels) on InsG3680^(+/+)^ mice as compared to InsG3680^(+/+)^ mice in the placebo control group exposed to air (air containing 21% oxygen gas, 1 ATA pressure levels) (Figure 1). All mice survived the procedures with no indications of irregular behavior or discomfort. At the end of the procedure (HBOT or placebo), 90 days-old (P90) mice were tested in behavioral tests that examined social behavior (three-chamber sociability and social novelty test), anxiety-like behavior (open field exploration test and elevated zero maze), and motor coordination (rotarod). Upon completion of the behavioral tests, mice were sacrificed and used for molecular and cellular studies (Figure 1).

**Figure 2:**
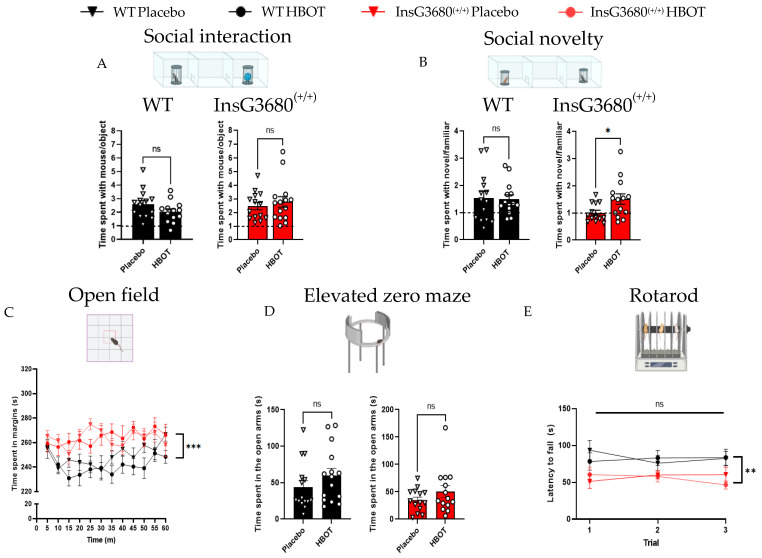
HBOT improves social novelty preference but not anxiety-like behavior or motor coordination in P90 InsG3680^(+/+)^ mice compared to placebo control mice. (**A**) No significant behavioral differences in the three-chambers social interaction test, as measured by a similar ratio between time spent in close interaction with a stranger mouse and an object in all experimental groups. (**B**) In the three-chambers social novelty test, mice from the InsG3680^(+/+)^ HBOT group showed a significantly higher preference for interacting with the novel mouse compared to the familiar mouse compared to mice from the InsG3680^(+/+)^ placebo group. No significant difference in the social novelty index was found between the WT HBOT group and the WT placebo group. (**C**) Time spent in the margins in the open field test, presented in time bins of 5 min. A significant main effect was found between the InsG3680^(+/+)^ and WT groups. Moreover, in the third time bin, the InsG3680^(+/+)^ HBOT group spent significantly less time in the margins than the InsG3680^(+/+)^ placebo group. A similar effect was found in the last time bin between the WT HBOT group and the WT placebo group. (**D**) Time spent in the open arms of the elevated zero maze test. HBOT did not affect the behavior in both genotypes. (**E**) In the rotarod test, no change was found in latency to fall throughout all three trials between HBOT and placebo in both genotypes. However, the WT groups have endured longer on the rotarod compared to InsG3680^(+/+)^ groups. ns—non significant, * *p* < 0.05, ** *p* < 0.01, *** *p* < 0.0005. Two-tailed *t*-test (**A**,**B**,**D**). 3-way ANOVA with repeated measures (**C**,**E**). Data are shown as mean  ±  s.e.m.

Because social behavior deficits are hallmark features of ASD, we studied whether HBOT has the potential to alleviate social behavior dysfunction. In the three-chambers sociability test, both the InsG3680^(+/+)^ HBOT group and the InsG3680^(+/+)^ placebo group spent significantly more time with the stranger mouse than with the object, as opposed to previous findings from this mouse model [7] (Figure 2A). Nonetheless, in the social novelty test, the InsG3680^(+/+)^ HBOT group showed significantly increased exploration time close to the novel mouse than the familiar mouse, whereas the InsG3680^(+/+)^ placebo group did not show any preference between them, similar to previously reported social novelty deficits [7] (Figure 2B).

Because elevated anxiety levels are known in ASD compared to typically-developed (TD) individuals, we next sought to characterize the effect of HBOT in ASD on anxiety-like behavior. In the open field test, no significant difference in overall exploration properties was found, except in the one-time bin (25 min), in which the InsG3680^(+/+)^ placebo group spent a significantly longer time in the margins compared to the InsG3680^(+/+)^ HBOT group (Figure 2C). In the elevated zero maze (Figure 2D) and the rotarod test (Figure 2E), no significant difference was found between the InsG3680^(+/+)^ HBOT group compared to the InsG3680^(+/+)^ placebo group.

### 2.2. HBOT Increases the Expression Level of Insulin-like Growth Factor 1 (Igf1) and Hif1a and Decreases Neuroinflammation in the Brain of InsG3680 Mouse Model for ASD

To seek neurobiological properties that could support the improved brain perfusion in the human patient and the improved behavior in our mouse model, we examined whether HBOT affects hypoxia and neuroinflammation properties. The indications of a hypoxic state and neuroinflammation in ASD [15,17,22] support the potential beneficial outcomes of HBOT as a potential treatment for the InsG3680 mouse model.

**Figure 3:**
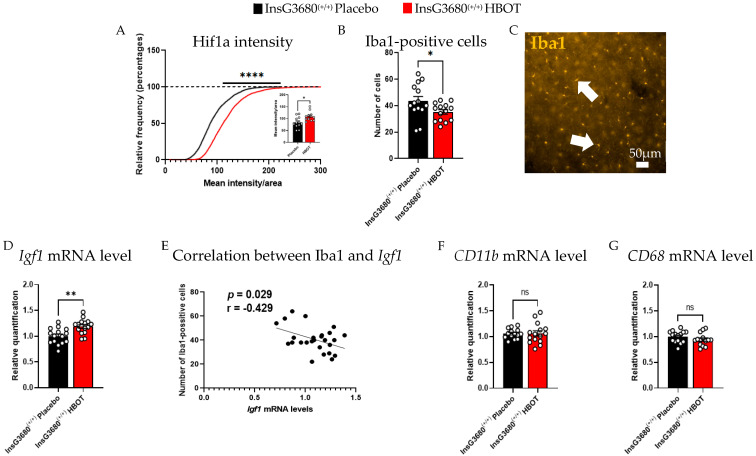
HBOT ameliorates neuroinflammation in P120 mice examined by molecular and cellular properties. (**A**) Cumulative distribution of the intensity of Hif1a divided by the area of the cell. Hif1a is significantly increased in the InsG3680^(+/+)^ HBOT group relative to the InsG3680^(+/+)^ placebo group (average intensity is divided by the averaged cell area). (**B**) The number of Iba1-positive cells is lower in the motor cortex of the InsG3680^(+/+)^ HBOT group compared to the InsG3680^(+/+)^ placebo. (**C**) Representative image of marked Iba1-positive cells marked by immunofluorescence staining. White arrows point to Iba1-positive cells. (**D**) The mRNA expression level of *Igf1* in the whole cortex of a 4-month-old is upregulated in the InsG3680^(+/+)^ HBOT group compared to InsG3680^(+/+)^ placebo. (**E**) A significant negative correlation between the number of Iba1-positive cells and *Igf1* expression levels in both InsG3680^(+/+)^ HBOT and InsG3680^(+/+)^ placebo. No significant difference was found in the mRNA expression level of (**F**) *CD11b* and (**G**) *CD68* in the whole cortex of 3 month-old InsG3680^(+/+)^ HBOT group compared to InsG3680^(+/+)^ placebo. ns—non significant, * *p* < 0.05, ** *p* < 0.01, **** *p* < 0.0001. Two-tailed *t*-test (**A**,**B**,**D**,**F**,**G**). Kolmogorov-Smirnov (**A**). Pearson’s correlation coefficient (**E**). Data are shown as mean  ±  s.e.m.

To examine whether HBOT impacted hypoxia, we examined protein levels of Hif1a. This protein is known to be upregulated during either hypoxia or hyperoxia and to function as a transcription factor to induce brain recovery. We found an increased intensity of Hif1a in the motor cortex of the InsG3680^(+/+)^ HBOT group compared to InsG3680^(+/+)^ placebo group (Figure 3A). This may indicate a recovery process of the tissue since Hif1a regulates angiogenesis processes and may alleviate neuroinflammation.

To characterize neuroinflammation properties, we examined the number of Iba1-positive cells and the expression level of key neuroinflammation-related transcripts in the whole cortex of InsG3680^(+/+)^ HBOT compared to InsG3680^(+/+)^ placebo. We found a reduced number of Iba1-positive cells in the motor cortex of the InsG3680^(+/+)^ HBOT group compared to InsG3680^(+/+)^ placebo group (Figure 3B,C). This result may imply a reduction in neuroinflammation following HBOT.

Since previous studies showed that IGF1 administration is effective in ameliorating ASD deficits [38,39] and could lead to a reduction in chronic neuroinflammation, we wanted to determine whether HBOT affects *Igf1* transcript expression level. We found upregulation in the expression level of *Igf1* in the whole cortex of the P120 InsG3680^(+/+)^ HBOT group compared to the InsG3680^(+/+)^ placebo (Figure 3D). Interestingly, we found a significant negative correlation between the number of Iba1-positive cells and *Igf1* expression levels in both InsG3680^(+/+)^ HBOT and InsG3680^(+/+)^ placebo (Figure 3E). No significant difference was found in the expression level of *CD11b* (Figure 3F) and *CD68* (Figure 3G), which are key neuroinflammation-related genes, as measured in the whole cortex of the P120 InsG3680^(+/+)^ HBOT group compared to InsG3680^(+/+)^ placebo group.

## 3. Discussion

In this study, we demonstrate for the first time that the core social behavioral deficit in ASD can be partially reversed by HBOT in a mouse model for ASD with *Shank3* gene mutation as in the human condition. As such, this study has the potential for translational and medical implications. It enables us to gain molecular and cellular mechanistic explanations that link behavioral improvement and brain changes induced by HBOT.

We found that HBOT improves the social behavior of adult InsG3680^(+/+)^ mice compared to InsG3680^(+/+)^ placebo control group mice, indicating that HBOT is effective in adults and not necessarily in the early postnatal developmental stage only. Specifically, HBOT improved social novelty preference, a subtype of social behavior tested in the three chambers social interaction test. The improved preference of a novel mouse compared to a familiar mouse in the social novelty test could also indicate improved cognition and memory. It may demonstrate that InsG3680^(+/+)^ treated mice remember the familiar mouse better than the InsG3680^(+/+)^ placebo group [40]. Nevertheless, it is important to note that the results in the three chambers social interaction test do not replicate the social preference deficit previously characterized in InsG3680^(+/+)^ mice compared to controls [7]. The lack of social deficit in our study might result from the repeated exposure of test mice to other test mice, coming from different cages and litters, daily, throughout the 2 months of the experiment. Also, the lack of improvement in anxiety-like behavior following HBOT might be related to a critical age at which the treatment should be given to reduce anxiety-like behavior to normal levels.

In search of molecular and cellular evidence that will explain how HBOT affected neurobiological properties, we characterized parameters related to the hyperoxic-hypoxic paradox [28]. Along the course of HBOT, the oxygen levels fluctuated, altering from normal (normoxia) to high (hyperoxia) oxygen levels. Those repeated fluctuations generate a new setting point where hyperoxia is interpreted as the new baseline and normoxia is interpreted as hypoxia with increased expression of HIF-1α. HIF-1α is upregulated and enters the nucleus to join an active complex with HIF-1β during hypoxia, while in normoxia, most HIF-1α is degraded in the cytoplasm [28,41]. A higher expression level of HIF-1α induces the expression of genes responsible for tissue regeneration, angiogenesis mediators such as VEGF, mitochondria metabolism, and anti-inflammatory processes [28]. In this study, we found increased expression of Hif-1α in the brain of the ASD mouse model following HBOT as compared to the InsG3680^(+/+)^ placebo group. To determine whether HBOT ameliorated the brain’s neuroinflammation, we studied the properties of microglia, resident macrophages of the brain. We found a reduced number of microglia (Iba1-positive cells) in the InsG3680^(+/+)^ HBOT group compared to the InsG3680^(+/+)^ placebo group. Previous studies indicated elevated immune response in the brain of autistic patients [42,43]; therefore, the reduced number of microglia due to HBOT may improve neural circuit activity [12] and reduce neuroinflammation in the brain of the mouse model for ASD. Nevertheless, microglia number does not necessarily represent the functionality of these cells. Hence, to gain more insight into the microglial properties on the molecular level, we studied the *Igf1* mRNA expression level following HBOT. IGF1 is a neurotrophic factor essential for central nervous system development [44,45,46,47], and microglia are an important source of IGF1. We measured an upregulation in the expression level of *Igf1* transcript in the whole cortex of the P120 InsG3680^(+/+)^ HBOT group compared to the InsG3680^(+/+)^ placebo group.

Previous studies found significantly reduced levels of IGF1 in the cerebrospinal fluid (CSF) of autistic children and infants compared to TD controls [48,49]. Of clinical relevancy, two studies in human patients with PMS showed improved social behavior, motor skills, and other behavioral symptoms following IGF1 administration [38,39]. In neurons derived from induced pluripotent stem cells (iPSCs) from ASD patients with *SHANK3* micro-deletion, IGF1 rescued synaptic transmission deficits in excitatory neurons [50]. Similarly, injection of IGF1 to *Shank3* deficient mice rescued abnormal motor skills and reversed major deficits related to excitatory synapse signaling [51]. Based on the above, IGF1 is a potential treatment for both syndromic and non-syndromic types of ASD [46,52,53,54]. Therefore, the upregulation of the *Igf1* expression level we measured following HBOT may lead to a cascading effect [55,56,57] that may improve brain functionality and behavioral outcomes.

Interestingly, when testing for a correlation between the number of Iba1-positive cells and the expression level of *Igf1,* we found a significant negative correlation between the two parameters. This correlation suggests that higher levels of *Igf1* are correlated with a potential reduction in neuroinflammation following HBOT and that *Igf1* may induce tissue repair, as was also found in previous studies [58,59,60]. Nonetheless, the results of our research show only a correlative and not a causative relationship.

Overall, our data suggest that HBOT is an effective treatment in the ASD genetic condition tested, reduces neuroinflammation, increases *Igf1* expression, and improves social behavior in adult mice.

The molecular and cellular effects we characterized following HBOT were examined 1 month after the HBOT session, suggesting a long-term effect of HBOT. Moreover, while HBOT was mainly studied in acute neurological conditions or chronic conditions in adulthood, our study shows the efficacy of HBOT in treating a genetic condition associated with chronic NDD starting from embryogenesis.

This research gives hope for the potential new biological intervention for a subtype of ASD, and future research should better dissect its long-term clinical effect. Looking forward, the use of treatments for ASD that are specifically proven to be effective on a specific genetic condition rather than on the phenotype of behavior hopefully marks the future of effective treatments for ASD. Since other subtypes on the ASD spectrum have overlapping pathophysiology, such as hypoperfusion, this study may be relevant to these other conditions as well.

## 4. Materials and Methods

### 4.1. Animal Work Statement

Terminology:

InsG3680^(+/−)^—mice that are heterozygous to the mutation.

InsG3680^(+/+)^—mice that are homozygous to the mutation.

WT—mice without the mutation on both chromosomes.

Breeding: To test the efficacy of HBOT on the InsG3680 mutation, InsG3680^(+/−)^ mice were crossed with InsG3680^(+/−)^ (Het-Het mating). InsG3680 and WT mice are with a C57 B6/S129 Sv mixed background. The resulting mice are either homozygous to the mutation (referred here as InsG3680^(+/+)^), heterozygous to the mutation (referred here as InsG3680^(+/−)^), or do not have the mutation (referred here as WT). The mutation occurs in all the cells of the body.

Housing: Mice of the same gender were contained in cages with 2–4 littermates with random genotypes. The environmental conditions were stable with 20–24 °C under a 12 h light/dark cycle (lights at 07:00 until 19:00), with food and water available at all times. All experimental procedures conformed to the guidelines of the Institutional Animal Care and Use Committee of Tel Aviv University, Tel Aviv, Israel. All efforts were made to minimize animal pain and suffering and the number of animals used.

### 4.2. HBOT

Treatment was performed on 1-month-old mice for 40 sessions for 2 months, 5 days a week, 1 h a day. A total of 30 mice were divided into 4 groups: InsG3680^(+/+)^ HBOT, InsG3680^(+/+)^ placebo, WT HBOT, and WT placebo. The HBOT group received 100% oxygen gas in 2 ATA pressure levels, while the placebo group received air (containing 21% oxygen) with 1 ATA pressure levels. The HBOT chamber was filled with 100% oxygen prior to compression for 5 min to enrich the oxygen in the chamber. Compression and decompression were carried out gradually to reduce the risks of changing pressures.

### 4.3. Behavioral Studies

Behavioral tests were performed and analyzed with the experimenter blinded to the type of treatment and genotype. Mice in the experiment went through habituation in the test room for 1 h before all tests. Each group of test mice was used for 4 behavioral tests and had at least 3 days between tests. The mice were tested at the age of 3 months (2 months after the start of the treatment).

#### 4.3.1. Social Preference Test

To perform the test, C57-black mice were ordered from the Jackson Laboratory with similar ages and body weights. Habituation for the stranger mice in the social preference test was performed by placing the mice inside an inverted wire cup for 30 min, 2 sessions per day for 3 consecutive days prior to the test. The test apparatus (65 cm long × 44 cm wide × 30 cm high) was divided into three sections (left, right—21 × 44 cm each, and center—21 × 22 cm), which were connected via a lever-operated door that was opened 5 cm from the floor. The object, stranger, and familiar mice were placed inside an inverted wire cup (10 cm high, diameter of bottom 10 cm, bars with 0.8 cm spacing), and on top of it, a weighted cup was placed to block the test mice from climbing over it. Following each trial, the wire cup was cleaned with ethanol and water. The test was comprised of three phases (15 min each): habituation, sociability, and social novelty. In the habituation phase, the test mouse was placed in the center and explored the environment of all three sections with the inverted wire cups empty. Next, in the sociability test, the tested mouse was placed in the center section with the doors closed while placing a stranger mouse and an object inside the wire cups of the left and right sections; Afterwards, the doors were lifted to allow the mouse to explore. Finally, in the social novelty test, the mouse was placed again in the center section with the doors closed while placing a novel mouse instead of the object, and afterward, the doors were lifted to allow the mouse to explore. The place of the object and stranger mouse were switched between trials to exclude place preference. Each of the stimulation mice were used only twice a day. Following the test, the experimenter analyzed the time spent with the object compared to a stranger mouse and the time spent with a familiar mouse compared to a novel mouse, blinded to treatment type, using the EthoVision XT 14.0.1326 software (Noldus Information Technology BV, Wageningen, The Netherlands).

#### 4.3.2. Open Field Exploration Test

Mice were placed in the center of a Plexiglas box (40 cm long × 40 cm wide × 30 cm high) for 1 h (one mouse in each box). Motor activity and exploration were videotaped and were measured by the time spent in the margins of the box while the experimenter was blind to the treatment type.

#### 4.3.3. Elevated Zero Maze

The maze contains 2 sections—open and closed arms (Height 60 cm). Mice were placed in the closed arms of the zero maze and explored for 5 min. Movement between the sections was videotaped and was measured by the time spent in the open arms, while the experimenter was blind to the type of treatment.

#### 4.3.4. Rotarod

Mice were placed on the apparatus of an accelerating rotarod, and motor coordination was measured by latency to fall. Each subject was tested three times with a time interval of 30 min between each test.

### 4.4. Brain Tissue Extraction

After treatment and behavioral experiments, male mice were deeply anesthetized with isoflurane. Consciousness was tested via a response to a pinch on the foot, and tissue samples from one ear were taken for genetic verification. Then, mice were perfused with 15 mL of ice-cold PBS solution, followed by brain dissection. The brain was separated into two hemispheres. One was kept in 4% paraformaldehyde (PFA) diluted in PBS for 4 consecutive days, and one was taken to a petri dish with PBS for dissection. The Cortex was separated from other brain tissue using cleaned surgical tools and a stereomicroscope (OLYMPUS, Kyoto, Japan). Cortices were placed in 200 μL of RNA-later solution (Invitrogen by Rhenium, Modi’in, Israel) and preserved at 4 °C for 24 h. RNA-later was taken out of the tubes, and samples were frozen at −80 °C. All tools and equipment were sterilized with ethanol and sprayed with RNAse inhibitor (RNase-ExitusPlus, Biological Industries, Israel).

### 4.5. RNA Isolation and Quantitative PCR (qPCR)

#### 4.5.1. RNA Extraction

Cortices were placed in safe-lock tubes with stainless steel beads and 200 μL of cold TRIzol reagent (Thermo Fisher Scientific, Waltham, MA, USA) and were homogenized in TissueLyser 2 (QIAGEN) for 40 s in 24,000 Hertz. After the tissue was fully homogenized, an additional 800 μL of TRIzol was added, and the samples were incubated at room temperature (RT) for 5 min. 200 μL of chloroform (Bio-Lab Ltd., Jerusalem, Israel) was added to each of the tubes, and they were shaken by hand for 15 min and incubated once more for 3 min at RT.

Then, samples were centrifuged for 20 min at 4 °C, 800× *g* (13,800 rpm) (Eppendorf Centrifuge 5430R, Eppendorf by Lumitron, Petah Tikva, Israel). The homogenate was separated into 3 layers-protein, DNA, and RNA, which was the top layer. The clear RNA layer was transferred to a new tube and was diluted with a proportion of 1:1 with isopropanol (Bio-Lab Ltd., Jerusalem, Israel). The tubes were manually shaken, incubated for 5 min at RT and centrifuged for 15 min at 4 °C, 800× *g* (13,800 rpm). After the centrifuge, the RNA was precipitated in the bottom of the tubs, isopropanol was taken out, and the remaining pellet was washed with 1 mL of 80% ethanol (Sigma-Aldrich, Rehovot, Israel) diluted in DEPC-treated water (Biological Industries, Kibbutz Beit-Haemek, Israel), followed by centrifugation for 15 min at 4 °C at 13,800 rpm.

This was repeated twice, while the ethanol was replaced the second time. All the ethanol was removed, and the tubes were placed upside down and opened on Kim wipes to let the remaining ethanol aspartate for approximately 30 min. 35 μL of DEPC-treated water was added to each sample, and then the sample was heated for 5 min at 60 °C. Finally, samples were pipetted to create homogenous concentration, measured using the Thermo Scientific NanoDrop One device (Thermo Fisher Scientific, USA), and kept frozen at −80 °C.

#### 4.5.2. Complementary Deoxyribonucleic Acid (cDNA) Preparation

RNA was diluted to a concentration of 20 ng/µL according to the sample’s original concentrations after RNA extraction. Reverse transcription was performed with random primers and the High-Capacity cDNA Reverse Transcription Kit (Thermo Fisher Scientific, Waltham, MA, USA). The protocol used with the C1000 Touch thermal cycler (Bio-Rad Laboratories, Hercules, CA, USA) was the following: 10 min at 25 °C, 120 min at 37 °C, 5 min at 85 °C, and a final step of 4 °C. cDNA was frozen at −20 °C.

#### 4.5.3. Real-Time PCR

mRNA levels were measured by Real-time PCR using the Fast SYBR Green PCR Master Mix (Thermo Fisher Scientific, Waltham, MA, USA) and the Bio-Rad CFX Connect Real-Time PCR Detection System (Bio-Rad Laboratories, Hercules, CA, USA). The protocol proceeded as follows: 20 s at 95 °C, 40 amplification cycles (3 s at 95 °C to denature, and 30 s at 60 °C to anneal and extend), and a melt curve: 60 °C for 5 s, and an increase of 0.5 °C every 5 s (including a plate read) until reaching 95 °C. mRNA levels were calculated based on the comparative cycle threshold (Ct) method [61]. To normalize each of the samples, the mRNA of glyceraldehyde 3-phosphate dehydrogenase (Gapdh) was also measured. Results are shown as fold change (FC) relative to the control group (InsG3680^(+/+)^ placebo). Primers were programmed in the lab and ordered from Hy Laboratories Ltd. (Rehovot, Israel). The final dilution of the primers was done with 10 mM in DEPC-treated water (see detailed sequence in Table 1).

### 4.6. Immunofluorescence Staining

Brains were extracted as described above and then sectioned at a 100 μm thickness using a vibratome (Leica Biosystems, Deer Park, IL, USA). Sections were stained using the free-floating method as follows: from each mouse, a section from the motor cortex was chosen (approximately bregma 0.5 mm, according to the mouse atlas) and was washed 3 times in 1 mL PBS for 5 min each. Then, sections were permeabilized with 1.2% Triton X-100 in PBS for 15 min. Following permeabilization, sections were washed in 1 mL PBS for 5 minutes each and blocked with 5% normal goat serum (NGS), 2% bovine serum albumin (BSA), and 0.2% Triton X-100 in PBS for 1 h. Afterward, sections were placed in a 96-well plate with 250 μL of primary antibodies diluted in blocking buffer (described above) overnight at 4 °C. The next morning, sections were washed in 1 mL PBS, once for 5 min and twice for 15 min each. Slices were then incubated with secondary antibodies conjugated with Alexa Fluor 488, 555, and 647 (1:1000; catalog nos. A11001, A21424; Invitrogen, Waltham, MA, USA) diluted in blocking buffer for 1 h. sections were washed in 1 mL PBS, once for 5 min and twice for 15 min each. Finally, for the mounting process on glass slides, VECTASHIELD Hardset Antifade Mounting Medium with DAPI (catalog nos. H-1500-10, Vector Laboratories, Newark, CA, USA) was used. Images were captured using a light microscope (IX-83, Olympus, Tokyo, Japan) with the experimenter blind to the treatment type. For quantification of cellular properties in the motor cortex, images were taken with ×10 or ×20 magnification according to the type of staining and analysis. Cell number and intensity were quantified manually using the ImageJ program.

Commercial antibodies used: anti Iba1 (1:500, catalog no. 234006, SYSY), anti Hif1a (1:400, catalog no. PA1-16601)

### 4.7. Statistical Analysis

Data are presented as the mean ± standard error of the mean (s.e.m.), as calculated by GraphPad Prism 9.4.1 for Windows (GraphPad Software, San Diego, CA, USA) *p*-values were calculated using Student’s *t*-test, 3-way repeated measures ANOVA, Kolmogorov-Smirnov and Pearson’s correlation coefficient, with *p* < 0.05 considered significant (* *p* < 0.05, ** *p* < 0.01, *** *p* < 0.0005, **** *p* < 0.0001). The normality of distributions and equality of variances were checked and addressed accordingly using the appropriate statistical analysis. Outliers were determined via the extreme studentized deviate (ESD) method (*p* < 0.05).

## Figures and Tables

**Table 1:** SYBR Green RT-PCR primers for mRNA quantification: *Mus musculus*.

Origin	Forward Sequence	Reverse Sequence
*Gapdh*	GCCTTCCGTGTTCCTACC	CCTCAGTGTAGCCCAAGATG
*Cd11b*	CTCTTGGCTCTCATCACTGCTG	GCAGCTTCATTCATCATGTCCT
*Igf1*	CAGCAGCCTTCCAACTCAATTAT	CGCCAGGTAGAAGAGGTGTGAA
*Cd68*	GGACAGCTTACCTTTGGATTCA	AAGGACACATTGTATTCCACCG
